# Editorial: Vulnerability and resilience in small island developing states

**DOI:** 10.3389/fpsyt.2024.1494503

**Published:** 2024-09-25

**Authors:** Michael H. Campbell, Maisha K. Emmanuel, Natalie S. Greaves, Myrna Lashley

**Affiliations:** ^1^ Faculty of Medical Sciences, The University of the West Indies, Bridgetown, Barbados; ^2^ Division of Trans-Cultural Psychiatry, Faculty of Medicine, McGill University, Montreal, QC, Canada

**Keywords:** mental health, climate change, resilience, small island developing states, Caribbean, Oceania

Small island developing states (SIDS) experience myriad geographic and socioeconomic vulnerabilities that affect the wellbeing of their communities. Although SIDS are not monolithic, commonly shared vulnerabilities include remoteness, small populations, disproportionate reliance on marine resources and imports, financial constraints and debt burden—and vulnerability to climate threat, which both exacerbates and is exacerbated by these existing pressures (1). Although climate researchers have acknowledged the disproportionate mental health impacts of acute (e.g., weather) and creeping (e.g., temperature and sea level increases) climate threats in SIDS (2), studies focused on mental health outcomes and interventions in SIDS are lacking. However, significant research efforts are now emerging (3–5). Indeed, since the inception of this Research Topic, the *Connecting Climate Minds* project has disseminated collaborative global and regional climate mental health research agendas, including for Oceania and Latin America and the Caribbean (6–8).

We appreciate the challenges faced by mental health researchers and clinicians in SIDS, who themselves are part of affected communities, subject to many of the same vulnerabilities as their neighbors—and who contribute to community resilience in their professional and personal roles. Further, many academic and healthcare institutions in SIDS operate in resource-limited settings that constrain production and dissemination of scholarship. Given those realities, providing a venue to feature the expertise and experiences of our SIDS colleagues was a principal motivation for organizing this collection of work, and we are pleased that the contributors are mostly mental health leaders from the Caribbean and Pacific regions, including researchers and clinicians from a broad range of relevant specialties and practice settings. The contributions themselves are similarly diverse, including empirical work, commentary, and a review paper. Taken together, the papers demonstrate the value of transdisciplinary and ecological approaches for conceptualizing and addressing mental health needs in SIDS.

The Research Topic presents qualitative findings from two recent Caribbean studies in the Commonwealth of Dominica and Republic of Trinidad & Tobago. LeBlanc et al. explore middle-aged Dominicans’ experiences and perceptions of severe weather events, explicating a range of negative physical and mental health outcomes, especially anxiety and burnout, as well the faith-based connections, community-based support, and individual coping behaviors that undergird resilience. Murphy et al. describe the lived experiences of youth in Trinidad & Tobago during COVID-19 lockdown restrictions. Their work situates the psychological stress experienced by young people navigating extraordinary disruption in broader context, connecting mental health outcomes with uncertain timelines for public health interventions, conflictual family dynamics in some households, separation from youth culture and social connections, educational fatigue, and anxieties about employment prospects. Both papers are useful for readers wishing to broaden their understanding of the psychological sequelae of recent public health crises in Caribbean SIDS. Importantly, they address a critical need to provide regionally relevant evidence to enhance public health preparedness as SIDS confront escalating climate threats (9).

Knight et al. provide a comprehensive review of attitudes and beliefs regarding psychosis among the Caribbean diaspora. Their valuable coverage provides a needed update of the literature and draws out key considerations for responsive research and practice with Caribbean people. The complex interrelationships of colonialism, global movement of populations, mistrust of mental health services, and cultural collectivism contextualize the experience of Caribbean people living with psychosis, as well as their families and communities. Dale et al. describe a study protocol that operationalizes a similar perspective to develop, implement, and assess a model of services for Aboriginal and Torres Strait islander youth in the Australian justice system. With an emphasis on co-design and community governance, they elaborate a research plan that is responsive in design and provision of services to support social and emotional wellbeing of young people in the cultural and geographic contexts of Melanesia and Australia. Finally, conceptual articles by Holdsworth et al. and Orbán present geographically and culturally informed recommendations for future climate health research in the Caribbean and Pacific regions, respectively. These pieces consider the vulnerabilities of SIDS in concert with the resilient aspects of cultures and local systems in framing the understanding and response to mental health impacts of climate change. Further, they recognize that the health impacts of climate change are embedded in dynamic socioeconomic and political systems that further affect public mental health outcomes (e.g., urbanization processes in South Pacific societies). Together, these contributions advocate for a broad approach to future climate mental health models for SIDS, grounded in a sense of place, informed by and responsive to local cultures, and inclusive of larger-scale geopolitical, social, and economic dynamics.

We are pleased that this Research Topic amplifies the voices of our researcher and practitioner colleagues in SIDS, whose scholarship demonstrates the importance of transdisciplinary and ecological frameworks for understanding the interrelationships among climate phenomena, socioeconomic factors, place, and mental health outcomes. This broader conceptualization is reflected in the emerging field of geopsychiatry, which emphasizes the intersections of mental health, economics, geography, climate change, migration, and geopolitics (10, 11). These themes feature heavily in this Research Topic, and we suggest that this integrated lens is useful for operationalizing future research as SIDS confront complex and escalating threats to physical and mental health. Future research should sharpen regional focus and expand scope of inquiry to promote the resilience of individuals and communities of SIDS, which are among the most vulnerable regions to global climate change.
